# Treatment patterns, healthcare resource utilization, and costs in patients with moderate‐to‐severe psoriasis treated with systemic therapy in Japan: A retrospective claims database study

**DOI:** 10.1111/1346-8138.16543

**Published:** 2022-08-10

**Authors:** Yayoi Tada, Hyunchung Kim, Dionysis Spanopoulos, Katsuyoshi Habiro, Katsuki Tsuritani, Yoshiyuki Yamada, Amartya Mandal, Yichen Zhong, Yusuke Hikichi

**Affiliations:** ^1^ Teikyo University Tokyo Japan; ^2^ Bristol Myers Squibb Tokyo Japan; ^3^ Bristol Myers Squibb Uxbridge UK; ^4^ Mu Sigma Bengaluru India; ^5^ Bristol Myers Squibb Princeton New Jersey USA

**Keywords:** cost of illness, health resources, medication adherence, practice patterns, psoriasis

## Abstract

The real‐world treatment landscape for patients with moderate‐to‐severe psoriasis receiving systemic therapies in Japan is not well understood. This study describes the demographic and clinical characteristics, treatment patterns, healthcare resource utilization, and psoriasis‐associated costs in these patients. This retrospective observational study used data from the Japan Medical Data Center database between January 2016 and December 2020. Eligible patients had a confirmed diagnosis of psoriasis, ≥1 claim for a systemic treatment of interest, medical history for ≥6 months, and follow‐up data for ≥12 months. Systemic therapies comprised biologics (tumor necrosis factor and interleukin inhibitors) and oral treatments (a phosphodiesterase‐4 inhibitor, immunosuppressants, and vitamin A). Patient demographics and clinical characteristics, treatment patterns, healthcare resource utilization, and costs were evaluated. The study identified 1770 patients satisfying all inclusion criteria. The mean age was 49.0 years, with 68% of patients aged 20–54 years. Overall, 90.6% and 9.4% of patients received oral medications and biologics as index treatment, respectively. Treatment patterns, healthcare resource utilization, and costs were assessed for treatments received by ≥20 patients (*n* = 1730). During the 12‐month follow‐up period, 1102/1730 patients (63.7%) discontinued index treatment, of whom 9.9% switched to alternative systemic treatments. The persistence rate was ≥70% for most biologics and <50% for oral systemic treatments. All 1730 patients had ≥1 all‐cause outpatient visit (2.0 visits per person per month) and hospitalization frequency was ≤0.01 per person per month. Persistent patients incurred inflation‐adjusted costs of Japanese Yen (JPY) 88 667 per person per month. Treatment switching was associated with an increase in total cost: JPY 128 039 per person per month after switching versus JPY 117 504 before switching. This study of Japanese patients with moderate‐to‐severe psoriasis demonstrated low persistence, high discontinuation, and low rates of treatment switching with systemic therapies. Switching was associated with increased total cost. These results indicate unmet needs for new treatments.

## 1 INTRODUCTION

Psoriasis (PsO) is a chronic autoimmune disorder affecting the skin and can manifest in the nails and joints.^1^ The prevalence of PsO in Japan is estimated to be 0.34%; it affects more than 560 000 individuals, with a greater prevalence in males (about 60%).^2^ In Japan, approximately 14% of patients with PsO are reported to have moderate‐to‐severe PsO.^3^

There are no formal treatment guidelines for PsO in Japan. The Japanese Dermatological Association (JDA) has provided detailed guidance on the use of biologics in PsO.^4^ However, there is no comprehensive treatment guideline that covers all systemic therapies. Nevertheless, treatment typically begins with topical agents, which may be followed by phototherapy. For moderate‐to‐severe cases, oral systemic treatments, such as with a phosphodiesterase‐4 (PDE‐4) inhibitor (apremilast), immunosuppressants (cyclosporin, methotrexate), vitamin A (acitretin, etretinate), or biologics may be subsequently prescribed.^5^ In Japan, apremilast was approved for use in PsO in 2016,^6^ whereas biologic therapies were launched between 2010 and 2019.^4^ Common biologics (and biosimilars) include tumor necrosis factor (TNF) inhibitors, such as adalimumab and infliximab; the interleukin (IL)‐12/23 inhibitor ustekinumab; IL‐23 inhibitors, such as guselkumab and risankizumab; and IL‐17 inhibitors, such as secukinumab, ixekizumab, and brodalumab.^1^, ^4^ Systemic therapies are often combined with phototherapy to attain better treatment efficacy.^7^, ^8^ A recent epidemiological survey by the Japanese Society for Psoriasis Research reported that between 2013 and 2018, in a population of patients with PsO, in 132 medical institutions,^9^ approximately 68.9% of patients with PsO received topical treatments, 9.1% of patients received phototherapy, 26.6% of patients were treated with oral systemic drugs, and 18.6% of patients were treated with biologics.^9^

Despite the wide variety of available treatments, the management of PsO has been associated with poor persistence and adherence worldwide.^10^ For oral systemic therapy, treatment adherence in France has been observed to be low, with at least 59% of patients discontinuing treatment in the first year.^11^ Real‐world evidence suggests that the 1‐year persistence rates of biologic treatments vary across demographics, with over 50% reported in Germany^12^, ^13^ and between 29% and 50% reported in the United States.^14^ Treatment discontinuation rates are typically high, ranging from 35% to 97%, depending on the treatment drugs used. However, upon discontinuation, only a small proportion of patients switch to alternative treatments (8%–34%).^14^, ^15^

Moderate‐to‐severe PsO is associated with considerably higher healthcare resource utilization (HCRU), with increased medication use and frequent outpatient visits compared with those in patients without PsO or psoriatic arthritis (PsA).^16^ These patterns have been well documented in the United States^17^ and Europe.^18^ Furthermore, several studies have demonstrated that increased HCRU is associated with disease severity.^19^, ^20^ However, the HCRU information for PsO in the Japanese population is scarce. Studies have assessed healthcare resource utilization for specific forms of PsO, such as generalized pustular PsO (GPP)^21^ and palmoplantar pustular PsO;^22^ however, few studies have investigated HCRU in PsO, with a focus on systemic therapy use. For example, a recent study by Okubo et al. compared HCRU between Japanese patients with GPP and plaque PsO and healthy controls, demonstrating greater HCRU, such as outpatient visits and hospitalization duration in patients with GPP and plaque PsO.^21^ Additionally, HCRU associated with apremilast use in Japan has also been documented.^23^ However, data on the direct impact of PsO on medical costs are limited. According to a study that used claims data from the Japan Medical Data Center (JMDC) from 2009 to 2016, the annual per patient total medical cost incurred by an average patient with PsO using biologics ranged between Japanese Yen (JPY) 2.2 and 3.4 million (approximately United States Dollar [USD] 17 000 to 27 000), demonstrating a high economic burden.^24^

The real‐world treatment landscape and the demographic and clinical characteristics of patients with moderate‐to‐severe PsO treated with systemic drugs in Japan are not well understood. Furthermore, to the best of our knowledge, no studies have been conducted to investigate the treatment costs and HCRU associated with both biologics and oral systemic therapies that are commonly prescribed for PsO in Japan.

The main objective of this study was to characterize treatment patterns, HCRU, and costs associated with systemic treatment in Japan, as well as patient demographics and clinical characteristics.

## 2 METHODS

### 2.1 Study design

This was a retrospective observational study that used the JMDC claims database^25^ to evaluate treatment patterns, HCRU, and costs in patients with moderate‐to‐severe PsO treated with systemic therapies in Japan. The data used in this study were collected between January 2016 and December 2020 (Figure S1). The study index period was between July 2016 and December 2019, and the index date was defined as the date of the first prescription of systemic therapy for PsO. Patients who had a PsO diagnosis and had initiated systemic treatments during the index period were followed up for 12 months.

### 2.2 Data source

The JMDC database comprises real‐world data since 2005 and covers epidemiological data and medical examination data from approximately 14 million people (as of February 2022)^25^ up to the age of 74 years.^26^ The database hosts anonymized patient records from more than 200 health insurance societies in Japan^26^ covering diagnoses; treatments and procedures received; inpatient, outpatient, and diagnosis procedure combination health insurance claims; and pharmacy claims, which allow traceability of patients across different providers from both hospital and general practice settings. Although details of the different treatments received by patients are available, the JMDC database does not capture reasons for any treatment that may have been discontinued.

### 2.3 Study ethics

This study utilized anonymized data from an existing claims database. Therefore, no ethics/institutional review board approval was necessary.

### 2.4 Patient selection criteria

Patients with a confirmed PsO diagnosis (International Classification of Diseases 10th Revision [ICD‐10] codes L40.0 and L40.9) and at least one claim for a systemic drug treatment during the index period were included. The first claim of systemic treatment during the index period was the index treatment, which should have been coupled with a PsO diagnosis at the index month.

Systemic treatments included both biologics and nonbiologic oral systemic agents, approved for treating moderate‐to‐severe PsO in Japan as given below.^1^, ^4^ Among biologics, treatment classes and associated drugs included the TNF inhibitors adalimumab, infliximab, and certolizumab pegol; the IL‐12/23 inhibitor ustekinumab; IL‐23 inhibitors guselkumab, risankizumab, and tildrakizumab; and IL‐17 inhibitors secukinumab, brodalumab, and ixekizumab. Oral systemic treatments included a PDE‐4 inhibitor (apremilast), immunosuppressants (cyclosporin and methotrexate), and vitamin A (etretinate). Eligible patients were required to be enrolled in the database for at least 6 months before the index month (pre‐index period) and for at least 12 months after the index month (follow‐up period). Patients were excluded if they had claims for ≥1 of the listed systemic treatments of interest in the pre‐index period. Patients <20 years of age at the index date and those with a confirmed diagnosis of inflammatory bowel disease, ankylosing spondylitis, rheumatoid arthritis, uveitis, juvenile arthritis, or atopic dermatitis in the pre‐index period were excluded from the analyses.

### 2.5 Outcome variables

Primary outcome variables included patient demographics at the index date; comorbidities and prior treatments, such as phototherapy, topical treatments, and other systemic drug therapies, including the use of antihistamines and antibiotics in the pre‐index period; and treatment patterns. Treatment patterns included the treatment type and clinical practice type (general practitioner [GP] or hospital [HP]) at index, and treatment discontinuation, persistence, and switching in the 12‐month follow‐up period. Treatment discontinuation, persistence, and switching were analyzed for only those treatment drugs that were received by ≥20 patients each at index. This thresholding was applied to increase the representativeness of estimates for individual treatment analyses, filtering out individual treatments represented by a low number of patients. The treatment drugs evaluated were adalimumab, ustekinumab, guselkumab, secukinumab, brodalumab, apremilast, cyclosporin, and etretinate. Infliximab, certolizumab pegol, risankizumab, tildrakizumab, ixekizumab, and methotrexate were not evaluated owing to a small sample size (<20 patients each). Treatment discontinuation was defined as having no prescription of the index systemic treatment for at least 60 days following the period covered by the last prescription.^22^, ^24^, ^27^, ^28^ The time to treatment discontinuation was defined as the time from the index date to the date of treatment discontinuation, i.e., the last day of the period covered by the last prescription. The systemic treatments of interest and their treatment intervals are listed in Table S1. Treatment switching, which was assessed among patients who discontinued the index treatment, was defined as having a prescription of a new treatment either during or within 60 days following the period covered by the last prescription. The persistence rate was determined using the Kaplan–Meier survival analysis and quantified as the proportion of patients who were persistent with treatment in the 12‐month follow‐up period. The time on treatment was defined as the time between the index date and the date of treatment discontinuation or data cutoff date. Persistent patients were those who were covered by the index treatment for 12 months, allowing for a gap of at most 60 days.

All secondary outcomes were analyzed for only those treatment drugs that were received by ≥20 patients each at index. Secondary outcomes assessed during the 12‐month follow‐up period included HCRU such as all‐cause outpatient visits and hospitalizations and phototherapy and topical treatment use, and treatment costs. The frequency of outpatient visits, the time interval between subsequent visits, and all‐cause hospitalization frequency and duration were analyzed. Costs included inpatient, all‐cause outpatient, and outpatient pharmacy costs, as well as total costs, all of which were assessed during the 12‐month follow‐up period. Cost before the treatment switch was estimated for the period between the index date and the date of the first switch (excluding the latter). Cost after treatment switch was estimated for the period beginning on the date of the first switch till the end of the 12‐month follow‐up period. Costs associated with treatment persistence or switching are reported for only those treatment classes where ≥20 patients persisted with or switched from index treatment during the 12‐month follow‐up period, respectively. All outcomes, except treatment persistence, are reported for treatment classes. Persistence is reported individually for each medication.

As an exploratory outcome, the incidence of PsA during the 12‐month follow‐up period was also assessed.

### 2.6 Statistical analyses

All data were analyzed in the total patient population and by treatment classes (treatment persistence was assessed for individual drugs as well). Categorical variables are reported using counts (*n*) and frequencies (%). Continuous variables are reported using descriptive statistics, such as median, mean, and standard deviation (SD). The 95% confidence intervals (CIs) are reported wherever applicable. All‐cause outpatient visits, all‐cause hospitalizations, and phototherapy frequency are reported per person per month (PPPM). All costs are reported in JPY as the ratio of the respective total cost to the accumulated number of person‐months and have been adjusted for inflation in Japan based on the calendar year average of the Consumer Price Index using the year 2020 as reference. All analyses were conducted using SAS software, version 9.4.

## 3 RESULTS

### 3.1 Patient disposition

**FIGURE 1:**
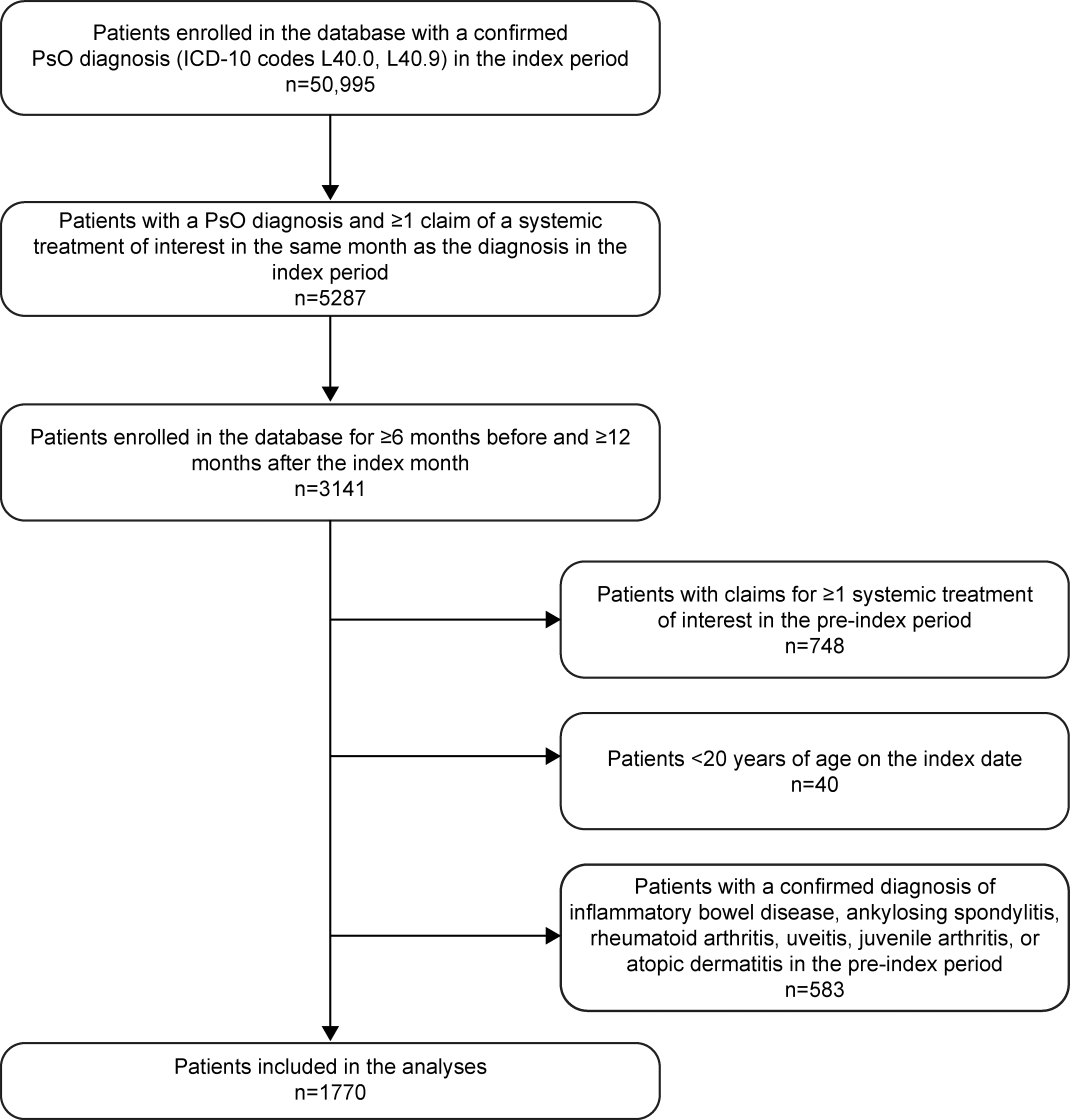
Patient disposition. ICD‐10, International Classification of Diseases 10th Revision; JMDC, Japan Medical Data Center; *n*, number of patients; PsO, psoriasis.

Of all the patients enrolled in the JMDC database, 50 995 had a confirmed diagnosis of PsO (per ICD‐10 codes L40.0 and L40.9) during the index period. The final cohort meeting all eligibility criteria comprised 1770 patients (Figure 1). Treatment patterns and HCRU and costs were analyzed for only those treatment drugs that were received by ≥20 patients each. Consequently, only 1730 patients were assessed.

### 3.2 Demographics and index treatment characteristics

In the population of 1770 patients who met the study criteria, the mean (SD) age of patients was 49.0 (10.5) years and 74.2% were male. Of these patients, 1203 (68.0%) belonged to the age group of 20 to 54 years. A total of 64.1% and 35.9% of patients were treated in GP and HP settings, respectively. For index treatments, oral systemic treatments were used by 90.6% of patients, with PDE‐4 inhibitor being the most common (53.8%), whereas biologics were used by 9.4% of patients. Oral systemic treatments were more commonly prescribed in the GP setting, whereas biologics were more commonly prescribed in hospitals. Comorbidities of interest such as cardiovascular disease, type 2 diabetes mellitus, metabolic syndrome, obesity, and depression were documented in the pre‐index period in 547 patients (30.9%), and the distribution was similar across patients receiving biologics and oral systemic treatments. Overall, 77.9%, 27.1%, and 14.0% of patients had a history of topical, systemic drug, and phototherapy treatments, respectively (Table 1).

**TABLE 1:** Demographics, clinical characteristics, and prior treatments

Category	Total	TNF inhibitor	IL‐12/23 inhibitor	IL‐17 inhibitor	IL‐23 inhibitor	PDE‐4 inhibitor	Immunosuppressant	Vitamin A
Total, *n* (%)	1770 (100.0)	32 (1.8)	24 (1.4)	78 (4.4)	32 (1.8)	953 (53.8)	388 (21.9)	263 (14.9)
Age, years								
Mean (SD)	49.0 (10.5)	47.1 (8.71)	47.4 (11.6)	47.4 (11.8)	48.8 (9.0)	48.7 (10.6)	47.9 (10.4)	52.9 (9.3)
Age group, years, *n* (%)								
20–34	178 (10.1)	2 (6.3)	4 (16.7)	13 (16.7)	3 (9.4)	106 (11.1)	41 (10.6)	9 (3.4)
35–44	358 (20.2)	9 (28.1)	6 (25.0)	12 (15.4)	5 (15.6)	195 (20.5)	95 (24.5)	36 (13.7)
45–54	667 (37.7)	14 (43.8)	7 (29.2)	29 (37.2)	16 (50.0)	355 (37.3)	149 (38.4)	97 (36.9)
55–64	465 (26.3)	6 (18.8)	5 (20.8)	21 (26.9)	8 (25.0)	244 (25.6)	82 (21.1)	99 (37.6)
≥65	102 (5.8)	1 (3.1)	2 (8.3)	3 (3.9)	0 (0.0)	53 (5.6)	21 (5.4)	22 (8.4)
Gender, *n* (%)								
Male	1313 (74.2)	24 (75.0)	20 (83.3)	62 (79.5)	24 (75.0)	732 (76.8)	265 (68.3)	186 (70.7)
Female	457 (25.8)	8 (25.0)	4 (16.7)	16 (20.5)	8 (25.0)	221 (23.2)	123 (31.7)	77 (29.3)
Clinical setting, *n* (%)								
GP	1134 (64.1)	2 (6.3)	1 (4.2)	8 (10.3)	2 (6.3)	717 (75.2)	246 (63.4)	158 (60.1)
HP	636 (35.9)	30 (93.7)	23 (95.8)	70 (89.7)	30 (93.7)	236 (24.8)	142 (36.6)	105 (39.9)
Comorbidities, *n* (%)								
Total	547 (30.9)	10 (31.3)	9 (37.5)	32 (41.0)	11 (34.4)	276 (29.0)	114 (29.4)	95 (36.1)
CVD	450 (25.4)	7 (21.9)	7 (29.2)	28 (35.9)	7 (21.9)	225 (23.6)	91 (23.5)	85 (32.3)
T2DM	108 (6.1)	1 (3.1)	2 (8.3)	6 (7.7)	4 (12.5)	56 (5.9)	24 (6.2)	15 (5.7)
Metabolic syndrome	1 (0.1)	0 (0.0)	0 (0.0)	0 (0.0)	0 (0.0)	1 (0.1)	0 (0.0)	0 (0.0)
Obesity	20 (1.1)	1 (3.1)	1 (4.2)	1 (1.3)	1 (3.1)	11 (1.2)	1 (0.3)	4 (1.5)
Depression	79 (4.5)	3 (9.4)	1 (4.2)	5 (6.4)	1 (3.1)	39 (4.1)	20 (5.2)	10 (3.8)
Prior treatments, *n* (%)								
Phototherapy	247 (14.0)	4 (12.5)	6 (25.0)	9 (11.5)	4 (12.5)	160 (16.8)	29 (7.5)	35 (13.3)
Other systemic drug therapy^a^	479 (27.1)	9 (28.1)	7 (29.2)	19 (24.4)	9 (28.1)	226 (23.7)	141 (36.3)	68 (25.9)
Topical treatment	1379 (77.9)	31 (96.9)	22 (91.7)	64 (82.1)	24 (75.0)	748 (78.5)	291 (75.0)	199 (75.7)

*Note*: TNF inhibitors include adalimumab, infliximab, and certolizumab pegol. IL‐12/23 inhibitor includes ustekinumab. IL‐23 inhibitors include guselkumab, risankizumab, and tildrakizumab. IL‐17 inhibitors include secukinumab, ixekizumab, and brodalumab. PDE‐4 inhibitor includes apremilast. Immunosuppressants include methotrexate and cyclosporin. Vitamin A includes etretinate.

Abbreviations: CVD, cardiovascular disease; GP, general practitioner; HP, hospital; IL, interleukin; *n*, number of patients; PDE‐4, phosphodiesterase‐4; SD, standard deviation; T2DM, type 2 diabetes mellitus; TNF, tumor necrosis factor.

^a^ Includes antihistamines and antibiotics.

### 3.3 Treatment patterns

#### 3.3.1 Treatment discontinuation and switching

During the 12‐month follow‐up period, 1102/1730 patients (63.7%) discontinued index treatment (Table 2). A total of 109/1102 (9.9%) patients who discontinued treatment had switched to a second systemic treatment; 90% of the patients did not initiate any other systemic treatment of interest within the following 60 days. Among patients receiving biologics as index treatment, the greatest discontinuation rate was observed in patients receiving the TNF inhibitor (72%), whereas the lowest discontinuation rate was observed in patients receiving an IL‐12/23 inhibitor (12.5%). Overall, 22.2% of the patients receiving TNF inhibitors switched treatments upon discontinuation, with oral systemic treatments being the most common second‐line treatment (Table 2), whereas the switch rate was ≤25% for all IL treatments. For oral systemic agents, the discontinuation rate ranged from 55.8% to 81.4%, with switch rates ranging from 6.5% to 16.8% in patients who discontinued index treatment (Table 2). Patients using immunosuppressants and vitamin A were more likely to switch to a PDE‐4 inhibitor (60% of cyclosporin discontinuers and 57.1% vitamin A discontinuers), whereas patients receiving a PDE‐4 inhibitor mostly switched to biologics (60%) (Table 2).

**TABLE 2:** Discontinuation and switching rates

Treatment class	Total number of patients, *n* (%)	Discontinuation rate, *n* (%)	Switching rate^a^, *n* (%)	Most common second‐line treatment
Total	1730 (100)	1102 (63.7)	109 (9.9)	
TNF inhibitor	25 (1.4)	18 (72.0)	4 (22.2)	Other oral systemic therapy
IL‐12/23 inhibitor	24 (1.4)	3 (12.5)	0 (0.0)	
IL‐17 inhibitor	60 (3.5)	30 (50.0)	6 (20.0)	Another IL‐17 inhibitor
IL‐23 inhibitor	28 (1.6)	4 (14.3)	1 (25.0)	IL‐17 inhibitor
PDE‐4 inhibitor	953 (55.1)	532 (55.8)	43 (8.1)	Biologics (60%); other oral treatments^b^ (40%)
Immunosuppressant	377 (21.8)	307 (81.4)	20 (6.5)	Apremilast
Vitamin A	263 (15.2)	208 (79.1)	35 (16.8)	Apremilast

*Note*: TNF inhibitor includes adalimumab. IL‐12/23 inhibitor includes ustekinumab. IL‐23 inhibitor includes guselkumab. IL‐17 inhibitors include secukinumab and brodalumab. PDE‐4 inhibitor includes apremilast. Immunosuppressant includes cyclosporin. Vitamin A includes etretinate.

Abbreviations: IL, interleukin; *n*, number of patients; PDE‐4, phosphodiesterase‐4; TNF, tumor necrosis factor.

^a^ Switching rate is computed only among patients who discontinued the index treatment.

^b^ Includes cyclosporin and etretinate.

#### 3.3.2 Treatment persistence

**FIGURE 2:**
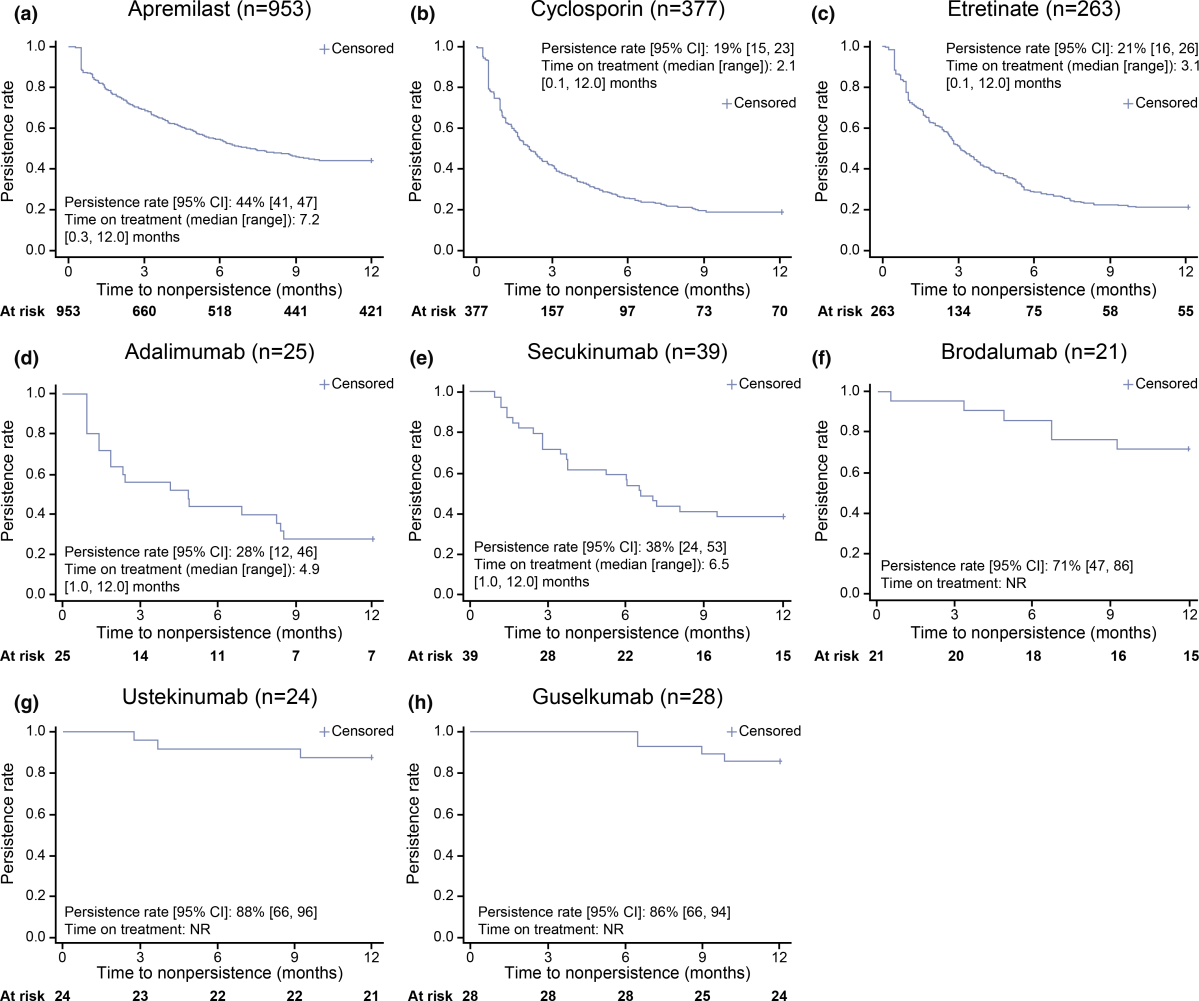
Persistence at 12 months by individual medication. (a–c) Persistence rate for patients receiving oral systemic treatments and (d–h) persistence rate for patients receiving biologics. CI, confidence interval; *n*, number of patients; NR, not reached.

The persistence rate at 12 months was generally high among patients receiving biologics (≥70% of patients receiving ustekinumab, brodalumab, and guselkumab), but low among patients receiving oral systemic treatments (<50%; Figure 2). Specifically, persistence rates [95% CI] were 44% [41, 47] for apremilast, 19% [15, 23] for cyclosporin, and 20% [16, 25] for etretinate. The time on treatment across all oral systemic treatments was <8 months (median [range]: apremilast, 7.2 [0.3, 12.0] months; cyclosporin, 2.1 [0.1, 12.0] months; and etretinate, 3.1 [0.1, 12.0] months), whereas, for most biologics, the median time on treatment could not be estimated as patients were censored at 12 months (Figure 2).

#### 3.3.3 Healthcare resource utilization

All 1730 patients who were assessed had all‐cause outpatient visits. In the 12‐month follow‐up period, the frequency of outpatient visits was 2.0 PPPM, with a mean (SD) duration between visits of 14.5 (18.8) days (Table 3). Among biologics and oral systemic treatments, the shortest time interval between outpatient visits was observed in patients receiving IL‐17 inhibitors (mean [SD]: 12.3 [17.5] days) and PDE‐4 inhibitor (mean [SD]: 14.0 [17.6] days), consistent with the relatively higher frequency of visits (IL‐17 inhibitor: 2.3 PPPM; PDE‐4 inhibitor: 2.1 PPPM) (Table 3). Across all systemic treatments, 125 patients had all‐cause hospitalizations, and in the study population, the overall frequency was low at ≤0.01 PPPM; however, among patients who had ≥1 hospitalization, the overall mean (SD) hospitalization duration across all treatment classes was 12.8 (18.8 days), ranging from 2 days to 58.3 days depending on the index treatment (Table 3). Overall, 0.18 PPPM phototherapy treatments were recorded. Phototherapy use during follow‐up was the highest in patients receiving PDE‐4 inhibitor as index treatment (*n* = 229), followed by patients receiving vitamin A (*n* = 55) and immunosuppressants (*n* = 27). Topical treatment during follow‐up was recorded in 93.5% of all patients and was most common among those with oral systemic index treatment (PDE‐4 inhibitor, 96.2%; immunosuppressant, 89.1%; vitamin A, 97.7%) (Table 3).

**TABLE 3:** Healthcare resource utilization during the 12‐month follow‐up period

	All‐cause outpatient visits (*n* = 1730)		All‐cause hospitalizations (*n* = 125)		Phototherapy	Topical treatment
	Frequency, [95% CI], (PPPM)	Duration between visits, Mean (SD), days	Frequency, [95% CI], (PPPM)	Duration, Mean (SD), days^**a**^	Frequency, [95% CI], (PPPM)	*n* (%)
Total	2.0 [2.0, 2.0]	14.5 (18.8)	0.01 [0.01, 0.01]	12.8 (18.8)	0.18 [0.17, 0.19]	1617 (93.5)
TNF inhibitor	1.7 [1.6, 1.9]	17.0 (19.0)	0.00 [0.00, 0.02]	3 (0.0)	0.00 [0.00, 0.02]	22 (88)
IL‐12/23 inhibitor	1.4 [1.2, 1.5]	21.7 (25.4)	0.01 [0.00, 0.03]	58.3 (49.5)	0.00 [0.00, 0.00]	21 (87.5)
IL‐17 inhibitor	2.3 [2.2, 2.4]	12.3 (17.5)	0.01 [0.00,0.01]	6.0 (6.2)	0.00 [0.00,0.01]	44 (73.3)
IL‐23 inhibitor	1.7 [1.6, 1.9]	17.0 (18.3)	0.00 [0.00, 0.02]	2 (0.0)	0.00 [0.00, 0.00]	20 (71.4)
PDE‐4 inhibitor	2.1 [2.0, 2.1]	14.0 (17.6)	0.01 [0.00, 0.01]	9.2 (19.0)	0.25 [0.24, 0.26]	917 (96.2)
Immunosuppressant	1.8 [1.8, 1.8]	15.7 (21.1)	0.01 [0.01, 0.02]	14.1 (15.1)	0.04 [0.04, 0.05]	336 (89.1)
Vitamin A	2.0 [2.0, 2.1]	14.2 (19.8)	0.01 [0.01, 0.02]	15.4 (16.1)	0.22 [0.21, 0.24]	257 (97.7)

*Note*: TNF inhibitor includes adalimumab. IL‐12/23 inhibitor includes ustekinumab. IL‐23 inhibitor includes guselkumab. IL‐17 inhibitors include secukinumab and brodalumab. PDE‐4 inhibitor includes apremilast. Immunosuppressant includes cyclosporin. Vitamin A includes etretinate.

Abbreviations: CI, confidence interval; IL, interleukin; *n*, number of patients; PDE‐4, phosphodiesterase‐4; PPPM, per person per month; SD, standard deviation; TNF, tumor necrosis factor.

^a^ For patients having at least one hospitalization.

#### 3.3.4 Psoriasis treatment costs

**FIGURE 3:**
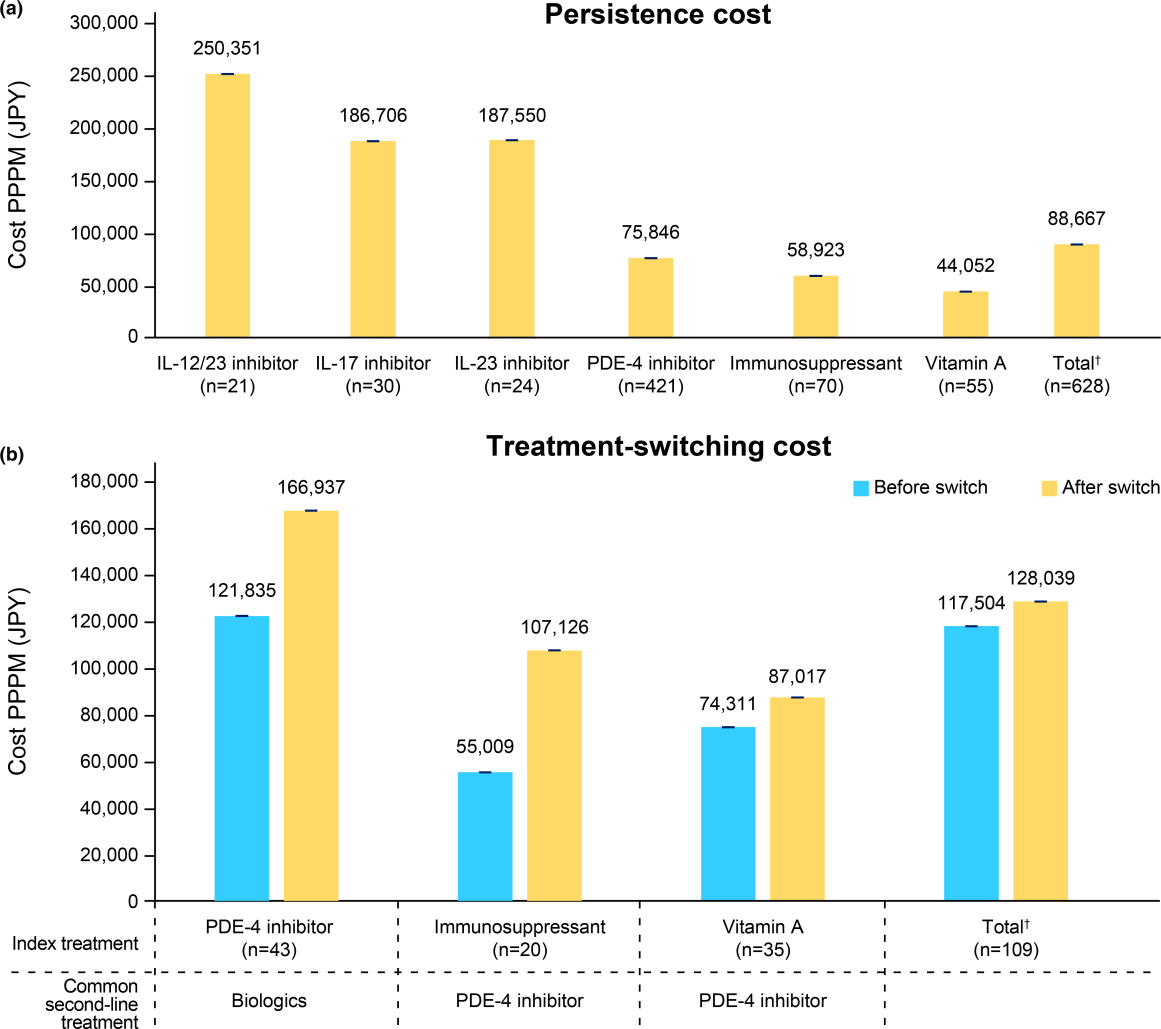
Cost associated with persistence and switching. (a) Cost for patients persisting on index treatment for 12 months and (b) cost for patients who switched to other treatments in the 12‐month follow‐up period. IL, interleukin; JPY, Japanese Yen; *n*, number of patients; PDE‐4, phosphodiesterase‐4; PPPM, per person per month. ^†^Includes biologics received by < 20 patients. Error bars indicate 95% confidence intervals.

Overall, patients persistent with index therapy for 12 months had total adjusted costs of JPY 88667 PPPM (95% CI, [88 661, 88 674]). Costs were highest among patients receiving biologics compared with those receiving oral systemic treatments (Figure 3a). Among patients persistent with their index treatment, treatment with vitamin A was associated with the lowest total costs [95% CI] (JPY 44 052 [44 036, 44 068] PPPM). Switching to a second‐line treatment after discontinuation of index treatment was associated with an increase in total healthcare costs. Across all treatment classes, the total medical cost [95% CI] PPPM after switching from index treatment was JPY 128 039 [128 015, 128 063] compared with JPY 117 504 [117 472, 117 536] before switching (Figure 3b). At least 20 patients receiving index treatments with immunosuppressants, PDE‐4 inhibitor, and vitamin A switched to other systemic treatments of interest. Among these patients, costs after switching treatments were greatest for those who switched from a PDE‐4 inhibitor to other treatments (Figure 3b).

#### 3.3.5 Incidence of PsA

Of all the 1770 patients receiving systemic treatments, 95 (5.4%) had a recorded PsA diagnosis during the 12‐month follow‐up period. Of these 95 patients, 19 had been newly diagnosed in the 12‐month follow‐up period and the remaining 76 had a pre‐index PsA diagnosis.

## 4 DISCUSSION

This study assessed the treatment patterns, HCRU, and costs among patients initiating systemic therapy for moderate‐to‐severe PsO in Japan.^25^ In this study, most patients belonged to the age group of 25 to 54 years. Oral medications were most commonly prescribed for the initiation of systemic treatment, including a PDE‐4 inhibitor, immunosuppressants, and vitamin A, and were more commonly prescribed in the GP setting. The results of our study showed generally poor treatment persistence, with high discontinuation rates and low post‐discontinuation switching rates with current oral systemic treatment and some biologics. Treatment switching was associated with increased total healthcare costs, suggesting an economic burden. A substantial unmet therapeutic need exists for patients with moderate‐to‐severe PsO in Japan.

The current study highlights that a majority of the patients belonged to the age group of 20 to 54 years, perhaps because the JMDC database adequately covers the working‐age group with information primarily based on claims from salaried workers and their families up to the age of 74 years from middle‐to‐large–sized insurance companies. Previous studies demonstrate the common age of PsO onset in Japan to be between 10 and 50 years,^29^ with an average age of 38.5 years.^30^ Furthermore, we observed that oral systemic treatments were prescribed more in the GP setting, whereas biologics were prescribed more in the HP setting. This finding is in keeping with the current JDA guidelines to administer biologics in hospitals.^4^

Our results demonstrate a high discontinuation rate with all systemic treatments (63.7%) within 12 months of index treatment initiation, with only 9.9% of these patients who discontinued systemic treatment initiating subsequent therapy within 60 days. A real‐world analysis conducted in patients with PsO in France showed that the 12‐month persistence rate for conventional oral systemic therapy was low (25%) and that 66% of patients did not initiate a new course of systemic treatment.^31^ Moreover, our findings are consistent with those in other reports from the United States, in which treatment switching after discontinuation of index treatment ranged from 8%^14^ to 34%.^15^ In accordance with earlier studies,^15^, ^32^ among the biologics, the highest discontinuation rate in this study was observed with adalimumab (TNF inhibitor) and the lowest with ustekinumab (IL‐12/23 inhibitor). Compared with biologics, oral systemic treatments such as PDE‐4 inhibitor and immunosuppressants were associated with a higher discontinuation rate in this study. Our findings are consistent with existing real‐world evidence from Japan, reporting a 1‐year drug survival rate for apremilast to be between 46% and 53%.^6^, ^33^ The switching patterns observed in the study were different from the conventional treatment progression pyramid plan used by clinicians in Japan.^5^ Typically, treatment for PsO begins with topical agents and subsequently phototherapy. For patients who do not respond to these treatments, systemic therapy is recommended. Patients may first be prescribed treatment with oral medications starting with vitamin A or a PDE‐4 inhibitor, followed by immunosuppressants. If still found unresponsive, patients may be prescribed biologics.^5^ However, our study results indicate that patients receiving apremilast most commonly switched directly to biologics, and those receiving immunosuppressants switched to apremilast. Treatment discontinuation is commonly associated with the worsening of symptoms, leading to poor outcomes for patients.^34^ The reasons for treatment discontinuation and the observed switching dynamics were not investigated in this study and should be explored in future real‐world evidence studies.

In this study, persistence during the 12‐month follow‐up period was variable across biologics, with high persistence observed with ustekinumab (88%) and low persistence observed with adalimumab (28%). Our findings are consistent with a real‐world study in Japan that demonstrated a 12‐month persistence rate of approximately 80% for ustekinumab and 46% for adalimumab.^24^ Several studies in Europe have also reported similar results—high persistence rates for biologics (>50%), with the greatest 1‐year persistence reported for ustekinumab.^28^, ^35^ Compared with biologics, in the current study, patients treated with oral systemic treatments demonstrated poor persistence, with a <50% persistence rate and a maximum time on treatment of approximately only 7 months. Our findings support those of a recent study assessing treatment persistence with oral systemic treatments for moderate‐to‐severe PsO in the United States, which demonstrated that the 12‐month persistence rate for apremilast was approximately 32.1%.^36^

Despite the long intervals for most biologic treatments (>1 month) and the availability of long‐term prescription policies (maximum of 3 months) in Japan, we observed frequent all‐cause outpatient visits of 2 PPPM in patients with PsO treated with systemic agents in this study, indicating a high HCRU. Our study also demonstrates the common use of combination therapy for moderate‐to‐severe PsO. Specifically, in all oral systemic treatment cohorts, nearly 90% of patients were taking topical treatment during the 12‐month follow‐up period, and phototherapy frequency ranged from 0.22 to 0.25 PPPM among patients treated with PDE‐4 inhibitor and vitamin A. Combining oral systemic treatments with phototherapy or topical treatments has been observed to improve treatment outcomes in moderate‐to‐severe PsO, demonstrating consistent trends with those reported in the literature.^8^, ^37^ Comorbidities such as PsA and associated treatments have also been shown to increase HCRU and related costs.^38^ In our study, 5.4% of patients had a recorded PsA diagnosis during the 12‐month follow‐up period. Further studies are warranted to understand the impact of combination therapies (e.g., phototherapy) and comorbidities (e.g., PsA) on HCRU and cost‐related outcomes for patients with PsO in Japan.

In this study, among patients who persisted with index treatment at 12 months, treatment costs associated with biologics were greater than those receiving oral systemic treatments, an anticipated finding that has also been reported in other studies.^39^, ^40^ Moreover, the cost associated with biologics ranged from JPY 2.2 to 3.2 million (approximately USD 17000 to 25 000) per patient per year, consistent with a previous finding using similar data from the JMDC database, for the period between 2009 and 2016.^24^ Our results demonstrate that treatment switching was associated with an increase in total costs, with a difference of JPY 10 535 (approximately USD 85) PPPM. A retrospective study in southern Italy assessing costs associated with biologic treatment for PsO reported a significant increase of 2680 Euros (approximately USD 3000) in yearly treatment cost per patient in patients who switched from the index treatment compared with those who did not.^41^ More recently, in the United States, Wu et al. demonstrated a significant increase of USD 1261 and USD 754 PPPM in patients who switched from apremilast or biologics (adalimumab, ustekinumab, etanercept, secukinumab, infliximab, and ixekizumab) to other treatments, respectively.^40^ In this study, the increment in total cost after treatment switching was assessed for treatment classes in which ≥20 patients switched from index treatment and included oral systemic treatments. Biologics constituted the most common second‐line therapy for patients previously initiated on PDE‐4 inhibitor, leading to an increase in cost by JPY 45102 (approximately USD 364) PPPM. Consistent with previous studies,^39^, ^40^ switching from oral systemic treatments to biologics can significantly raise treatment expenditure because the medical cost associated with biologic treatments is greater than that for oral systemic treatments. The economic burden of treatment switching may have precluded patients from seeking alternative therapies after discontinuation of their index therapy. A low rate of treatment switching after discontinuation despite a high discontinuation rate underscores the need for long‐term and more sustainable treatment options.

Certain limitations of this study must also be noted. The sample size of patients receiving biologics is small. Therefore, all associated results must be interpreted with caution. Additionally, the precise reasons for treatment discontinuation, such as adverse events, lack of efficacy, and voluntary withdrawals, could not be determined and should be explored in future investigations. These limitations highlight the need for additional studies for a more comprehensive understanding of treatment patterns. The results of this study may not be generalizable to all systemic treatment‐naïve patients with PsO in Japan, as data for patients aged ≥75 years in the JMDC database are limited. However, the database adequately covers the age group wherein PsO is more prevalent in Japan.^2^ Moreover, as the database does not include information on disease severity or disease activity, upon discussion with a dermatology practitioner and based on previous studies,^1^, ^4^ we have defined moderate‐to‐severe PsO by patient use of common systemic therapies. Therefore, disease severity may not have been accurately captured. Furthermore, as the JMDC database only includes month‐level outpatient data, the all‐cause costs and HCRU have been reported in this study. Despite these limitations, to the best of our knowledge, this is the first report demonstrating real‐world treatment patterns, cost, and HCRU, using a large claims database covering both biologics and oral systemic treatment for patients with PsO in the last 5 years.

Although many treatment possibilities exist, patients with PsO are often undertreated or untreated. Studies in the United States have indicated that 32.2% of patients with moderate‐to‐severe PsO were untreated^42^ and 21.5% to 29.5% of patients only received topical treatments.^43^ Moreover, the Multinational Assessment of Psoriasis and Psoriatic Arthritis survey found that <10% of patients were using systemic therapy.^44^ This study highlights the treatment patterns, HCRU, and costs associated with moderate‐to‐severe PsO in patients initiating systemic treatment in Japan. Despite the availability of several treatment options and the greater use of oral systemic treatments, low persistence and high discontinuation rates coupled with low post‐discontinuation switching rates highlight the unmet needs for patients with moderate‐to‐severe PsO in Japan. The reasons for poor adherence and persistence to treatments are not fully understood. Possible reasons for treatment discontinuation and lower rates of switching could include patient reluctance in taking injections, particularly biologics. Therefore, further studies assessing patients' treatment preferences and reasons for discontinuation would provide more insight into patient needs. Treatment switching after discontinuation of index systemic treatment also imposes a meaningful economic burden. More effective and sustainable treatment strategies, including oral/pre‐biologic treatments, are needed for patients with moderate‐to‐severe PsO in Japan.

## CONFLICT OF INTEREST

YT reports grants from AbbVie, Boehringer Ingelheim, Bristol Myers Squibb, Eisai, Eli Lilly, Kyowa Kirin, LEO Pharma, Maruho, Mitsubishi Tanabe Pharma, Sun Pharmaceutical Industries Limited, Taiho Pharmaceutical, Torii Pharmaceutical, and UCB Pharma; consulting fees from AbbVie, Boehringer Ingelheim, Bristol Myers Squibb, Eli Lilly, Janssen, Maruho, Novartis Pharma, Taiho Pharmaceutical, and UCB Pharma; and honoraria from AbbVie, Boehringer Ingelheim, Bristol Myers Squibb, Eisai, Eli Lilly, Janssen, Kyowa Kirin, LEO Pharma, Maruho, Mitsubishi Tanabe Pharma, Novartis Pharma, Sun Pharmaceutical Industries Limited, Taiho Pharmaceutical, Torii Pharmaceutical, and UCB Pharma. HK, KH, KT, YY, YZ, and YH are employees of and report profit of stock or stock options from Bristol Myers Squibb. DS is an employee of Bristol Myers Squibb. AM is an employee of Mu Sigma.

## DATA AVAILABILITY

Restrictions apply to the availability of these data, which were used under license for this study. The data that support the findings of this study are not publicly available and cannot be shared with external researchers.

## Supporting information


Appendix S1
