# Supplementary material for: Treatment patterns, healthcare resource utilization, and costs in patients with moderate‐to‐severe psoriasis treated with systemic therapy in Japan: A retrospective claims database study
Source: J Dermatol. 2022 Aug 10;49(11):1106–17. doi: 10.1111/1346-8138.16543 (PMC9804179; doi:10.1111/1346-8138.16543)
Supplement: Supplementary file 1 — Appendix S1 [file JDE-49-1106-s001.pdf]

SUPPLEMENTARY MATERIAL

SUPPLEMENTARY FIGURE 1 Study design

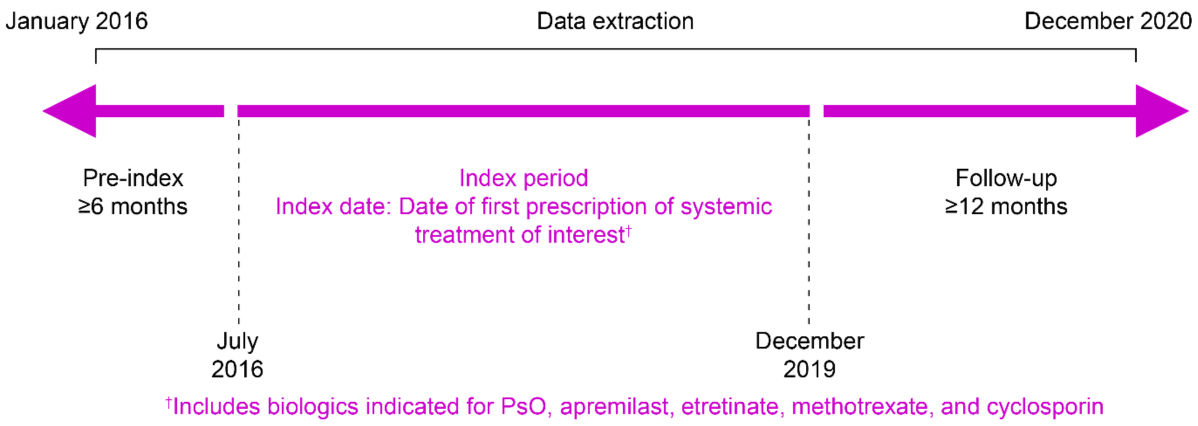

PsO, psoriasis.

SUPPLEMENTARY TABLE 1 Systemic treatments of interest

**Drug class**	**Drug generic name**	**Treatment interval, days (Upon maintenance dosing schedule)**
**TNF inhibitor**	Infliximab, including biosimilars	56
	Adalimumab, including biosimilars	14
	Certolizumab pegol	14
**IL-12/23 inhibitor**	Ustekinumab	84
**IL-17 inhibitor**	Secukinumab	28
	Ixekizumab	28
	Brodalumab	14
**IL-23 inhibitor**	Guselkumab	56
	Risankizumab	84
	Tildrakizumab	84
**PDE-4 inhibitor**	Apremilast	NA
**Immunosuppressants**	Cyclosporin	NA
	Methotrexate	NA
**Vitamin A**	Etretinate	NA

IL, interleukin; NA, not applicable; PDE-4, phosphodiesterase-4; TNF, tumor necrosis factor.
